# Validation of Antiobesity Effects of Black Soybean Seed Coat Powder Suitable as a Food Material: Comparisons with Conventional Yellow Soybean Seed Coat Powder

**DOI:** 10.3390/foods10040841

**Published:** 2021-04-13

**Authors:** Yuuki Moriyasu, Chiho Fukumoto, Maki Wada, Erika Yano, Hiroshi Murase, Masatoshi Mizuno, Nobuhiro Zaima, Tatsuya Moriyama

**Affiliations:** 1 Department of Applied Biological Chemistry, Graduate School of Agriculture, Kindai University, 3327-204 Naka-machi, Nara 631-8505, Japan; mrmr874g@gmail.com (Y.M.); aiampochi@gmail.com (C.F.); wdkdgradsch@gmail.com (M.W.); pyonchangoods@yahoo.co.jp (E.Y.); zaima@nara.kindai.ac.jp (N.Z.); 2 Otsuka Foods Co., Ltd., Biwako Research Institute, 1-11-1 Karasaki, Otsu-shi, Shiga 520-0106, Japan; Murase.Hiroshi@otsuka.jp (H.M.); Mizuno.Masatoshi@otsuka.jp (M.M.); 3 Agricultural Technology and Innovation Research Institute, Kindai University, 3327-204 Naka-machi, Nara 631-8505, Japan

**Keywords:** obesity, metabolic syndrome, lifestyle-related diseases, polyphenols, anthocyanins, adipose tissue, adipocytokine, health functionality, functional food material

## Abstract

In this study, we fed obese model mice black soybean seed coat powder (BSCP) and evaluated the antiobesity effects. As a control, normal yellow soybean seed coat powder (YSCP) was used. C57BL/6J, a high-fat diet-induced obesity model mouse, was fed a high-fat diet containing BSCP or YSCP (20% fat) to induce obesity. The results showed that in the BSCP group, it caused significant suppression of body weight gain and suppression of white adipose tissue weight compared with the YSCP group. Moreover, it significantly decreased serum leptin levels, which correlated with visceral fat mass, and increased antidiabetic adipocytokine and adiponectin levels. Therefore, this suggests the pigmented components contained in BSCP have an antiobesity effect in obese model mice. It is suggested that this material, which can be prepared without extraction with an organic solvent and is suitable for use as a food material, could be a functional food material with a practicable antiobesity effect.

## 1. Introduction

Recently, an increase in the number of patients with diabetes and cardiovascular diseases associated with obesity has become a social problem; thus, the search for food ingredients that suppress insulin resistance and hyperglycemia caused by obesity and elucidation and practical application of their mechanisms of action have been widely attempted [1].

Soybean has been widely used as a source of oil and protein since ancient times, but its physiological functions, such as lifestyle-related disease prevention effects, have been noted in recent decades [2]. Soybean contains three major nutrients (proteins, carbohydrates, and fats), and also a variety of functional components, including soy peptides, isoflavones, linoleic acid, linolenic acid, plant sterols, lecithin, saponins, and other nutrients, such as dietary fiber, minerals, and vitamins [2,3,4,5]. Many of these functional components have been studied; however, there are a few components that have been recognized as new functional components and are expected to be developed in the future [2,3,4,5].

Black soybean is a kind of soybean and is widely known as a nutritious health food in Oriental countries [6]. The characteristic difference between black and yellow soybeans is that the black soybean seed coat contains abundant polyphenol components [7,8]. The pigment component of the seed coat comprises polyphenols, which mainly include anthocyanins and proanthocyanidins [9,10]. These polyphenols have a wide array of bioactivities, such as antioxidant, antiatherosclerosis, anticarcinogenic, and anti-inflammatory properties [11,12,13,14]. Among them, proanthocyanidins, which account for most of the polyphenol composition, are known as condensed tannins [15], and are found in many plants other than black soybeans, including grape seeds, apples, persimmons, and pine bark, and have a range of bioactivities, such as antiarteriosclerosis, antidiabetes, and antiobesity. Antihyperlipidemia, antimutagenicity, anti-inflammatory, and various physiological activities have also been reported [16,17,18].

The absorption and metabolism of proanthocyanidins are determined by their chemical structure; the lower the degree of polymerization of proanthocyanidins, the higher the absorption rate into the body and the higher the bioavailability [19]. Moreover, compared to pure aglycones, flavonoid glycosides are more bioavailable [20,21]. Proanthocyanidins contained in black soybean are low-molecular-weight compounds with a degree of polymerization of 1 to 30 [22]; low-molecular-weight oligomers, such as procyanidin B2, C1, and cinnamtannin A2, are the main constituents [15], which are absorbed by the small intestine and are distributed throughout the body.

Therefore, in this study, the pigment components contained in black soybean seed coats were used as actual food ingredients and whether the pigment components of black soybean seed coats exerted effective antiobesity effects was examined by feeding obese mice black soybean seed coats that were finely crushed to a powder (BSCP) as feed, without extracting them with organic solvents, and comparing their effects to mice fed yellow soybean seed coat powder (YSCP).

## 2. Materials and Methods

### 2.1. Materials

Cornstarch, casein, sucrose, cellulose, American Institute of Nutrition (AIN)-93M mineral mix, and AIN-93M vitamin mix were purchased from Oriental Yeast Inc. (Tokyo, Japan). Methionine and choline chloride were purchased from Nacalai Tesque Co., Ltd. (Kyoto, Japan). Lard was purchased from the Marine Foods Corporation (Tokyo, Japan). Measurement kits for levels of serum glucose (Glc), triacylglycerol (TG), total cholesterol (T-chol), high-density lipoprotein (HDL) cholesterol, and phospholipids (PLs) were purchased from Fujifilm Wako Co., Ltd. (Osaka, Japan). Enzyme-linked immunosorbent assay (ELISA) kits for mouse leptin, mouse adiponectin, and mouse resistin were purchased from R&D Systems, Inc. (Minneapolis, MN, USA). All other chemicals used in this study were of the highest available purity.

### 2.2. Preparation of Soybean Seed Coat Powder

**Figure 1:**
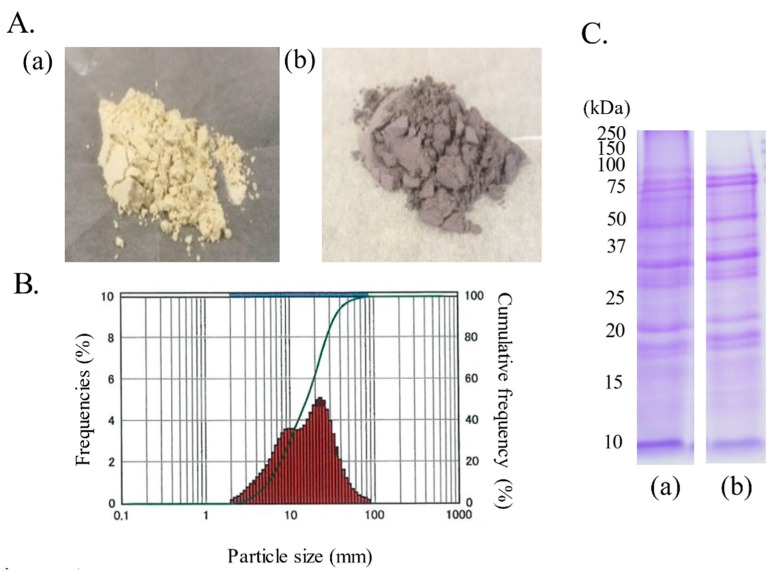
Characterization of black soybean seed coat powder (BSCP) and yellow soybean seed coat powder (YSCP). (**A**) Appearance of BSCP (**a**) and YSCP (**b**). (**B**) Particle size of BSCP. (**C**) Protein patterns of BSCP (**a**) and YSCP (**b**).

This study used finely ground soybean seed coat powder produced by spray drying (Figure 1). These food materials were provided by Otsuka Food Co., Ltd. (Osaka, Japan).

### 2.3. Component Analysis of Fine Soybean Seed Coat Powder

YSCP or BSCP was subjected to ingredient analysis to examine the amounts of major nutritional components (Table 1). The protein patterns of these powders were analyzed by sodium dodecyl sulfate-polyacrylamide gel electrophoresis (SDS-PAGE) and Coomassie brilliant blue (CBB) staining (Figure 1).

### 2.4. Animal Experiments

Male C57BL/6J mice (5 weeks old) were obtained from Japan SLC (Shizuoka, Japan) and maintained in an air-conditioned room at 20 ± 2 °C with a 12-h light-dark cycle. During the acclimation week, distilled water and experimental diet chow (MF, Oriental Yeast, Tokyo, Japan) were provided ad libitum. The mice were subsequently divided into two groups (YSCP group: *n* = 10, BSCP group: *n* = 11) so that their mean body weights were approximately the same and fed a high-fat diet containing 20% (*w*/*w*) lard for 10 weeks, containing YSCP (8 g/100 g total diet) or BSCP (8 g/100 g total diet). Dietary composition and energy intake are described in Table 2. Dietary intake measurements and dietary changes were performed six times per week, and total food intake was calculated during the experimental period. The mice were weighed twice a week. After 10 weeks of feeding, mice were fasted for 4 h and sacrificed by blood sampling under anesthesia with sodium pentobarbital. The liver and white adipose tissue (WAT, peritesticular fat, mesenteric fat, and perirenal fat) were retrieved, washed with 0.9% (*w/v*) saline, weighed, and stored at −80 °C until use. This study was approved by the Kindai University Animal Experiment Committee (Approval No. KAAG-25-002).

### 2.5. Analysis of Serum Parameters

After blood collection, serum was recovered by centrifugation at 3000× *g* for 10 min at 4 °C. Levels of Glc, TG, T-chol, HDL-cholesterol, and PLs were each measured according to the manufacturer’s instructions for the measurement kits. Serum leptin, adiponectin, and resistin levels were measured according to the manufacturer’s instructions for the commercially available ELISA kits.

### 2.6. Analysis of Liver Lipids

Total lipids were extracted from livers harvested at dissection using the methods described by Folch et al. [23]. Briefly, extracted livers were homogenized, and organic solvents were used to extract total lipids. TG and T-chol levels were measured using a commercial measuring kit, as described above.

### 2.7. SDS-PAGE and Western Blot Analysis

Proteins in peritesticular fat lysates were separated by SDS-PAGE and transferred onto polyvinylidene difluoride membranes (Immobilon™-P, Merck Millipore, Billerica, MA, USA). Antibodies against uncoupling protein-2 (UCP-2) (GTX132072, GeneTex, Inc., Irvine, CA, USA), β-actin (MAB1501, Merck Millipore), horseradish peroxidase (HRP)-labeled anti-mouse IgG (Thermo Fisher Scientific Inc., Waltham, MA, USA), and HRP-labeled anti-rabbit IgG (Thermo Fisher Scientific Inc.) were used for Western blotting. All immunoreactive proteins were detected using ECL™ Western Blotting Detection Reagents (GE Healthcare, Buckinghamshire, UK).

### 2.8. Statistical Analysis

Differences between the means of the two groups were analyzed using the Student’s *t*-test. For all studies, a *p* value < 0.05 was considered significantly different.

## 3. Results

### 3.1. Average Body Weight Transition, Food Intake, Body Weight Gain, and Feed Efficiency

**Figure 2:**
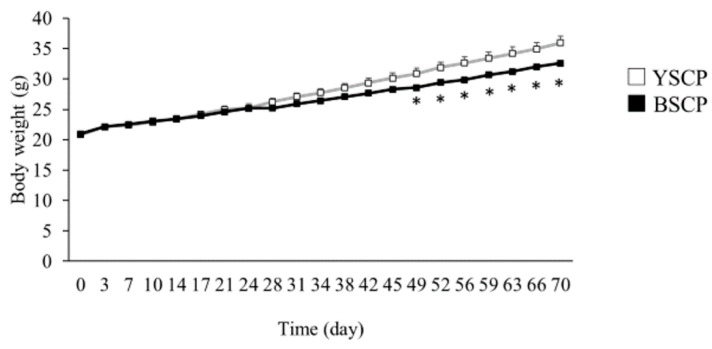
Effect of yellow soybean seed coat powder (YSCP) or black soybean seed coat powder (BSCP) on the average body weight of mice fed high-fat diets for 10 weeks. ■, mice given BSCP; □, mice given YSCP. Values are presented as the mean ± standard error (SE, *n* = 10 or 11). Differences between groups were compared using Student’s *t*-test. An asterisk indicates a significant difference (*p* < 0.05).

Treatment of mice with a high-fat diet containing BSCP or YSCP resulted in significantly lower body weights of mice fed BSCP than those fed YSCP on day 49 of the test period. In addition, the body weight of mice fed BSCP was significantly lower than that of mice fed YSCP in all subsequent periods (Figure 2). In contrast, the total food intake of mice was not significantly different between the two groups; thus, it was concluded that the difference in the type of soybean in the sample had no effect on food intake. Body weight gain and feed efficiency (body weight gain/total food intake) were also significantly lower in mice fed BSCP than that in mice fed YSCP (Table 3). Thus, consumption of BSCP prevented weight gain induced by a high-fat diet compared to consumption of YSCP.

### 3.2. Various Fat Weights

Peritesticular fat weight and perirenal fat weight were significantly reduced in mice fed BSCP, while mesenteric fat tended to be lower but was not significantly different between the two groups (Table 3). 

These results indicated that BSCP ingestion prevented an increase in visceral fat induced by a high-fat diet compared to ingestion of YSCP.

### 3.3. Serum Analysis

There were no significant differences in Glc, TG, T-chol, HDL cholesterol, and PL levels between the BSCP and YSCP groups (Table 4).

Interestingly, serum adiponectin levels were significantly higher in mice fed BSCP than those in mice fed YSCP. In contrast, serum leptin levels were significantly lower in mice fed BSCP than those in mice fed YSCP. In addition, serum resistin levels tended to be lower in mice fed BSCP than those in mice fed YSCP, although the difference was not significant (Table 4).

### 3.4. Liver Weights and Lipid Parameters

There was no significant difference in liver weights between the two groups (Table 5). TG and T-chol levels were not significantly different between the two groups (Table 5).

### 3.5. Western Blot Analysis

**Figure 3:**
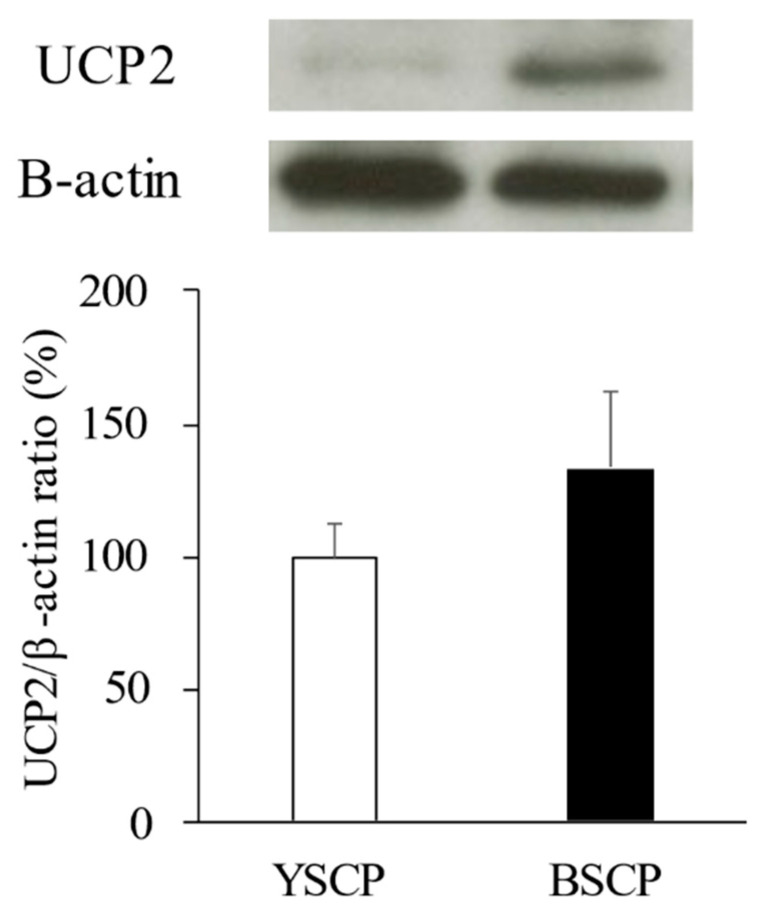
Effect of yellow soybean seed coat powder (YSCP) or black soybean seed coat powder (BSCP) on protein levels of UCP-2 in peritesticular fat of mice fed high-fat diets for 10 weeks. ■, mice given BSCP; □, mice given YSCP. Protein levels of UCP-2 are measured by western blot analysis. Values are presented as the mean ± standard error (SE, *n* = 10 or 11). Differences between groups were compared using Student’s *t*-test.

The expression of UCP-2 in peritesticular fat tended to be higher in mice fed BSCP than that in mice fed YSCP, but there was no significant difference between the two groups (Figure 3).

## 4. Discussion

In this study, the antiobesity effects of BSCP, which was developed for ease of application as a food material, were evaluated. A spray drying method was used to produce this fine powder. The spray-dry method is useful for continuous and high-volume productivity, and results in a cheaper cost because of lower product loss [24]. In addition, the particle size of the soybean seed coat powder used in this study is approximately 10–50 μm in diameter (Figure 1B). The use of such a fine-milled powder increases the surface area and reduces the feeling of scattering, which improves the absorption efficiency of the active ingredients into the body and reduces discomfort when mixing with other food materials.

Previous studies using black soybean seed coat extracts that were extracted with acidic water and ethanol have reported that black soybean polyphenols suppress obesity and promote the amelioration of glucose dysmetabolism [25]. Administration of black soybean seed coat extract to high-fat diet-dependent obese mice results in significant suppression of body weight and mesenteric fat weight and plasma glucose levels, and significantly increases insulin sensitivity [25].

Herein, the antiobesity effects were examined using the fine powder of the black soybean seed coat without performing a complicated and costly extraction process, with the aim of its practical application as an actual food material. To minimize the effects of components other than the pigments contained in the black soybean seed coat, this study was conducted using conventional YSCP as a control.

BSCP ingestion during a high-fat diet challenge significantly suppressed body weight transition, body weight gain, and feed efficiency without affecting total food intake in mice compared to mice fed YSCP. Relatedly, WAT, especially peritesticular and perirenal fat weights, showed significantly lower values. Thus, the suppressive effect of BSCP ingestion on body weight gain was hypothesized to be mainly due to the suppression of adiposity.

Leptin is an adipocytokine that is positively correlated with visceral fat mass [26,27]. Therefore, serum leptin levels were measured using ELISA, and in mice fed BSCP, serum leptin levels were significantly lower than those in mice fed YSCP. These results also support the reduction of visceral adipose mass in mice fed BSCP. However, as obesity progresses, adiponectin, an adipocytokine that exhibits beneficial functions, such as insulin sensitivity and vasoprotective effects, decreases [28,29]. In the current study, adiponectin levels were significantly higher in mice fed BSCP, which prevented the obesity-induced decrease in beneficial hormones. In addition, resistin, a “bad” adipocytokine involved in inflammatory and/or insulin resistance [30,31], also tends to be lower in mice fed BSCP. These changes in the secreted amounts of adipocytokines may be the result of a significant reduction in fat mass in mice fed BSCP. It was hypothesized that the pigmented components contained in BSCP, either directly or indirectly, acted on adipocytes and promoted adipocyte shrinkage, causing a significant increase in adiponectin secretion, a significant decrease in leptin secretion, and a tendency to lower resistin secretion.

In white adipocytes, UCP-2, which undergoes thermogenesis instead of ATP production, is involved in energy expenditure [32]. Polyphenols containing high concentrations of black soybean seed coat extracts significantly increase mRNA and protein expression of UCP-2 [25]. In this study, when UCP-2 protein expression in peritesticular fat is measured by Western blotting, it tends to be higher in mice fed BSCP, though this difference is not significant, as shown in Figure 3. It is thought that the improvement of energy metabolism by increasing the expression of UCP-2 promotes a decrease in white fat mass [32,33]. Therefore, since the polyphenols contained in the BSCP used in this study seem to have similar effects, it was presumed that, as in this previous study, improved energy metabolism resulting from elevated expression of UCP-2 prompted a reduction in white fat mass.

Moreover, procyanidins from cacao species exhibit obesity-suppressive effects by phosphorylating AMP-dependent protein kinase (AMPK) in high-fat diet-fed mice [34]. AMPK acts as a regulator to maintain the homeostasis of various cellular energetics and is involved in the regulation of the whole-body energy balance [35,36]. Adiponectin is known to phosphorylate and activate AMPK [37]. Thus, these findings also suggested that the pigmented components of BSCP might exhibit obesity-suppressive effects through the phosphorylation of AMPK. In the present experiments, the mechanism by which the pigmented components of BSCP were involved in obesity-suppressive effects and secretory regulation of adipocytokines were not determined and should be studied in the future.

In conclusion, this study showed that BSCP, which was prepared without manipulation, such as extraction from black soybeans, and was a form suitable for addition and application to foods, directly had antiobesity potency compared to ordinary YSCP. Food ingredients with this new antiobesity effect may be used as novel “seeds” for functional foods by mixing them with various foods.

## Figures and Tables

**Table 1:** Nutrients of yellow and black soybean seed coat powder.

	YSCP	BSCP
Energy (kcal/100 g)	253	240
Fat (g/100 g)	3.9	2.0
Protein (g/100 g)	13.5	9.7
Carbohydrate (g/100 g)	74.1	78.2
Sugar (g/100 g)	7.9	13.2
Dietary fiber (g/100 g)	66.2	65.0
Ash (g/100 g)	4.5	4.8
Moisture (g/100 g)	4.0	5.3

YSCP: yellow soybean seed coat powder; BSCP: black soybean seed coat powder.

**Table 2:** Chemical composition of experimental diets (g).

	YSCP		BSCP	
	Content (g)	Energy (kcal)	Content (g)	Energy (kcal)
Cornstarch	18.1	72.2	17.8	71.4
Casein	10.5	37.8	10.7	38.4
Sucrose	5.0	20.0	5.0	20.0
Cellulose	0.3	0.7	0.4	0.8
AIN-93M mineral mix	1.6	0.0	1.6	0.0
AIN-93M vitamin mix	0.5	0.0	0.5	0.0
Methionine	0.1	0.0	0.1	0.0
Choline chloride	0.1	0.0	0.1	0.0
Soybean powder	4.0	10.1	4.0	9.6
Lard	9.8	88.4	9.9	89.1
Water	50.0	0.0	49.9	0.0
Total	100.0	229.2	100.0	229.2

YSCP: yellow soybean seed coat powder; BSCP: black soybean seed coat powder; AIN: American Institute of Nutrition.

**Table 3:** Total food intake and body fat weights of mice.

		YSCP	BSCP
Total food intake (g)		473 ± 6.5	543 ± 19.7
Weight gain (g)		15.1 ± 0.81	11.6 ± 0.56 *
Feed efficiency (%)		3.20 ± 0.15	2.14 ± 0.06 *
Body fat weight (g)		3.07 ± 0.10	2.21 ± 0.09 *
	Peritesticular fat	1.85 ± 0.05	1.26 ± 0.03 *
	Perirenal fat	0.66 ± 0.25	0.49 ± 0.13 *
	Mesenteric fat	0.57 ± 0.28	0.46 ± 0.22
Body fat percentage (%)		8.44 ± 0.07	6.76 ± 0.07 *
	Peritesticular fat	5.06 ± 0.12	3.87 ± 0.07 *
	Perirenal fat	1.80 ± 0.43	1.50 ± 0.39
	Mesenteric fat	1.57 ± 0.03	1.39 ± 0.03 *

YSCP: yellow soybean seed coat powder; BSCP: black soybean seed coat powder. Feed efficiency is calculated as the weight gain as a percentage of the total food intake. Visceral fat weight as a percentage of the whole-body weight was calculated using the body weight on the last day of the diet. Values are presented as the mean ± standard error (SE, *n* = 10 or 11). Differences between groups were compared using the Student’s *t*-test. Asterisks indicate significant differences between different values within a row (*p* < 0.05).

**Table 4:** Blood serum parameters and adipocytokines levels in mice.

	YSCP	BSCP
Glucose (mg/dL)	308 ± 21	318 ± 24
Triglyceride (mg/dL)	71 ± 6	64 ± 11
Total cholesterol (mg/dL)	135 ± 6	132 ± 10
HDL-cholesterol (mg/dL)	103 ± 3	96 ± 5
Phospholipid (mg/dL)	169 ± 5	164 ± 3
NEFA (mEq/L)	1.15 ± 0.08	1.17 ± 0.10
Adiponectin (µg/mL)	7.71 ± 0.26	8.54 ± 0.26 *
Resistin (ng/mL)	105 ± 19.3	62.6 ± 14.5
Leptin (ng/mL)	12.8 ± 1.08	6.67 ± 0.67 *

YSCP: yellow soybean seed coat powder; BSCP: black soybean seed coat powder; HDL: high-density lipoprotein cholesterol; NEFA: non-esterified fatty acids. Values are presented as the mean ± standard error (SE, *n* = 10 or 11). Glucose, triglyceride, total cholesterol, HDL cholesterol, phospholipid, and NEFA were determined using an enzymatic assay kit. Adiponectin, resistin, and leptin levels were determined using enzyme-linked immunosorbent assay. Differences between groups were compared using the Student’s *t*-test. Asterisks indicate significant differences between different values within a row (*p* < 0.05).

**Table 5:** Liver weights and lipid parameters of mice.

	YSCP	BSCP
Liver weight (g/100 g bw)	3.87 ± 0.26	3.43 ± 0.05
Liver triglyceride (mg/liver g)	19.9 ± 5.9	17.7 ± 2.2
Liver total cholesterol (mg/liver g)	0.56 ± 0.15	0.56 ± 0.10

YSCP: yellow soybean seed coat powder; BSCP: black soybean seed coat powder. Values are presented as the mean ± standard error (SE, *n* = 10 or 11). Differences between groups were compared using the Student’s *t*-test.

## Data Availability

The data presented in this study are available on request from the corresponding author.
